# Polysubstance and Behavioral Addictions in a Patient with Bipolar Disorder: Role of Lifetime Subthreshold Autism Spectrum

**DOI:** 10.1155/2018/1547975

**Published:** 2018-02-22

**Authors:** Liliana Dell'Osso, Ciro Conversano, Martina Corsi, Carlo A. Bertelloni, Ivan M. Cremone, Barbara Carpita, Manuel G. Carbone, Camilla Gesi, Claudia Carmassi

**Affiliations:** Department of Clinical and Experimental Medicine, University of Pisa, Pisa, Italy

## Abstract

This case report draws attention to the potential relevance of undetected autism spectrum symptoms in a bipolar patient with high work functioning showing a peculiar addictive profile with impulsive and antisocial behaviors. A 23-year-old man with a diagnosis of Bipolar Disorder (BD) and Substance Use Disorder (SUD) was hospitalized at the Psychiatric Clinic of the University of Pisa for diuretics and *β*-2 adrenergic agonist abuse in a remission phase of benzodiazepines and substance abuse. He reported a history of behavioral addictions in the framework of a global high work functioning with particular skills in computer science. When assessed for adult autism spectrum symptoms, despite not fulfilling a DSM-5 diagnosis of Autism Spectrum Disorder (ASD), he reported a score of 93/240 at the Ritvo Autism and Asperger Diagnostic Scale (RAADS-r) and of 88/160 at the Adult Autism Subthreshold Spectrum (AdAS Spectrum), both indicative of ASD. We argue the possible role of adult subthreshold autism spectrum features, generally disregarded in adult psychiatry, in the peculiar addictive profile developed by this patient with BD that may deserve appropriate treatment.

## 1. Introduction

Mental disorders and substance abuse frequently cooccur with about 50% of individuals with severe and persistent mental disorders reporting Substance Use Disorders (SUD). High rates of psychiatric comorbidity among patients with SUD have been specifically reported for mood disorders, above all Bipolar Disorder (BD), but data are also available for psychotic, posttraumatic stress, anxiety, and eating disorders [1–6].

Some new perspectives, however, derive from most recent research on Autism Spectrum Disorders (ASD). Growing literature, in fact, indicates ASD subjects to show higher rates of comorbidity with anxiety, mood, psychotic, trauma, and stress-related disorders, as well as with suicidal behaviors. Consistently, increasing attention has been devoted to these milder forms that may be undetected or misdiagnosed [7, 8]. Current Diagnostic and Statistical Manual of Mental Disorder (DSM-5) ASD encompasses mild forms of autism, with normal or above average intelligence that may reach high levels of functioning, developing in some cases excellent abilities in restricted areas.

The emerging psychopathological challenge of ASD, particularly in the case of subthreshold, is that it may remain undiagnosed until adulthood or misdiagnosed because of other overlapping mental disorders; sometimes, in fact, ASD remains unrecognized even after the onset of these latter. Takara and Kondo reported 16% ASD prevalence rate among first-visit depressed adult patients, while Kato et al. reported rates as high as 7.3% of previously unrecognized ASD among suicide attempters hospitalized for inpatient treatment [9–12].

Recently, intriguing findings about a possible cooccurrence of ASD and SUD have been highlighted in adult patients. Up to date research, in fact, indicates a lifetime prevalence of SUD in about 11% to 29% of ASD patients, rising the question about the most appropriate treatment for both disorders once recognized at an early stage [13–15]. Interestingly, among ASD patients, early onset of SUD has not been associated with more severe disability scores than later onset one, suggesting the idea that a subgroup of patients with former SUD may have a higher level of functioning before the onset of SUD in comparison to those without lifetime SUD [16]. A qualitative study using in-depth interviews on 12 patients with comorbid SUD and ASD pointed out that substances might serve to solve some ASD-related problems in the short run while leading to negative consequences in the long run, such as difficulties with the structuring of daily life because of a lack of initiative [2]. Lalanne et al. reported two cases of high-functioning autism patients by using alcohol and psychostimulants to cope with their anxiety and to improve their cognitive abilities. According to these authors, alcohol use disorder might be even underdiagnosed among such patients, in virtue of the normalizing effect displayed by alcohol on social skills of these patients [17]. Consistently with these observations, a study conducted on 129 ASD or ADHD adults, with and without a history of SUD, proved that, with regard to the social skills level, patients with ASD and comorbid SUD showed less impairment than those without SUD [18].

The aim of this article was to explore the possible influence of undetected adult autism spectrum features on the particular addictive behaviors reported by a bipolar patient with comorbid SUD.

## 2. Case Presentation

### 2.1. Sociodemographic and Clinical Details

The patient (XY) is a single 23-year-old man, working as computer scientist in a technological company, who was admitted to our outpatient psychiatric clinic reporting a problematic use of alcohol and benzodiazepines (BDZ). In his past psychiatric history, he reported experiencing bullying at the beginning of the secondary school while showing very good marks at school. He started the use of substances (alcohol and tetrahydrocannabinoids on social occasions) at the age of 20 years old and rapidly moved to cocaine use, showing a fast progression, from a weekly use to a multiday use in less than a month. Cocaine use had been going on for six months without detrimental effect on social adaptation and stopped when the patient was uncovered by his parents. Subsequently, right after the interruption of the use of cocaine, he undertook gambling behaviors (scratch and win) with substantial loss of money. In that period he also presented a dysmorphic attitude and assumed anabolic steroids (testosterone, nandrolone). These episodes occurred during a period of six months predominantly characterized by elevated mood, which was followed, a few months later, by a clinical picture of internal tension and increased anxiety levels. For this reason, he was treated by his general practitioner with BDZ (delorazepam, bromazepam) which led to further mood swings complicated by the abuse of alcohol and high dose of BDZs.

At the age of 22 years old, the patient turned to a psychiatrist and started a psychopharmacological therapy, at first with antidepressants such as paroxetine and escitalopram, without benefits on both affective and addictive aspects. He was then prescribed valproic acid and disulfiram with partial improvement of the mood symptoms despite continuing binges of alcohol and benzodiazepines. Interestingly, he reported that he premeditated such binge episodes, which occurred every 15–20 days, not taking disulfiram.

At the age of 22 years old he was admitted to the Outpatient Unit of the Psychiatric Clinic of the University of Pisa (Italy), where he was diagnosed with BD type II in a patient with a problematic use of alcohol and a history of SUD. He received a psychopharmacological treatment based on mood stabilizers (lithium salts, valproic acid), disulfiram, clonazepam, and nalmefene, with a significant reduction of the mood fluctuations, cessation of alcohol consumption, and partial improvement of BDZ abuse. More recently he was admitted to the Inpatient Unit of the Psychiatric Clinic of the University of Pisa; consequently, an episode of diuretics (furosemide) and *β*-2 adrenergic agonists (clenbuterol) misuse lasted for two weeks, associated with an increase of the physical activity upon a period when he attended the local gym. He reported that diuretics self-administration would have served the increased concern about his physical appearance since his body dissatisfaction represented a limit in friendship. At the time of the admission, he showed underestimation of the medical risks to witch he was exposed.

The treatment strategy adopted led to a remarkable improvement in his clinical situation, including substantial mood stabilization, a reduction in anxiety, the prevention of withdrawal symptoms for alcohol and BDZ, cessation of alcohol use, reduction of craving for alcohol and BDZ, and improvements in social functioning. Relapses on fast-acting BDZ use continued to occur, but their frequency reduced. To date, the patient is still working as computer scientist in a technology company and has an optimum working adaptation, but he is poorly adapted to the social and leisure plan with no social circle and lack of confidence in social situations.

### 2.2. Assessment Instruments

The patient was assessed by means of the Structured Clinical Interview for DSM-5 Disorders (SCID-5), the Ritvo Autism and Asperger Diagnostic Scale (RAADS-r) [19], and the Adult Autism Subthreshold Spectrum (AdAS Spectrum) [20].

The RAADS-r is a modified version of the Ritvo Autism Asperger's Diagnostic Scale [21] including 80 items divided into four subdomains:* language and communication* (7 questions),* social relatedness* (39 questions),* sensorimotor and stereotypies* (20 questions),* circumscribed interests* (14 questions). The questions are designed for individuals with average IQ and above.

The AdAS Spectrum is a questionnaire developed within the framework of the international research network called* Spectrum Project* [22]. The instrument assesses the lifetime presence of the wide spectrum of manifestations associated with ASD, but which could be founded even in individuals who do not fulfill diagnostic criteria for a formal disease: in this regard, it was not developed to be a diagnostic instrument. The AdAS Spectrum allows evaluating a broader area of clinical and nonclinical traits. It includes 160 dichotomic questions (yes/no), grouped in seven domains:* childhood/adolescence, verbal communication, nonverbal communication, empathy, inflexibility and adherence to routine, restricted interests and rumination, hyper/hyporeactivity to sensory input*. The AdAS demonstrated an excellent reliability, with a Kuder–Richardson coefficient of 0.964 for the total score and above 0.80 for each single domain, except for the* empathy *and* hyper/hyporeactivity to sensory input *domains, which had, respectively, a coefficient of 0,762 and 0.794. The test-retest reliability was also demonstrated to be adequate (Intraclass Correlation Coefficients above 0.90). Moreover, AdAS total score was highly correlated with RAADS-14 and AQ total score, with a Pearson's *r* correlation coefficient, respectively, of 0.77 and 0.83, and each AdAS domain positively correlated with RAADS-14 and AQ (ranging from *r* = 0.58 to *r* = 0.79) [20].

By means of the SCID upon DSM-5 criteria, a diagnosis of BD type II and SUD emerged, while just one out of two criteria of ASD was satisfied.

A total score of 93 on 240 was reported at the RAADS-r as well as a total score of 88 on 160 at the AdAS Spectrum lifetime. Details on RAADS-r and AdAS are shown in Table 1.

## 3. Discussion

We presented the case of a 23-year-old man with BD type II and SUD in a peculiar addictive profile and a set of impulsive and antisocial behaviors in the context of high work functioning drawing attention to the potential relevance of undetected autism spectrum features. The patient presented a diagnosis of BD type II with prevalent hypomanic symptoms, which represent a risk factor for the development of SUD [23–25]. What is noteworthy in the present case is the onset of substance abuse as well as the wide range of substance and behaviors addictions, which included drugs without addictive power, such as diuretics and *β*-2 adrenergic agonists. The patient did not seem to present the sociodemographic profile of patients who usually show a rapid escalation of substance use: he is male, is Caucasian, has been engaged in substance use relatively later than youngest addicts, and does not belong to a community of deviant addicts, as he works as computer scientist and has a relatively poor social adaptation [24–28]. Other psychopathological details resulting in the context of the psychiatric assessment included a RAADS-r total score of 93 and an AdAS Spectrum total score of 88. Both are consistent with a diagnosis of subthreshold ASD. Furthermore, the RAADS-r showed high scores in two subdomains: language and communication with a score of 9 and social relatedness with a score of 54, where the threshold values for suspected ASD are 4 and 31, respectively. It is interesting that the patient showed scores consistent with ASD in the clusters related to social interactions but not in sensorimotor and stereotypies and circumscribed interests. According to a distinct perspective, the AdAS Spectrum identifies subthreshold features of ASD and it is noteworthy to underline that the patient showed a considerable number of positive items in almost all the domains of the questionnaire except for the hyper/hyporeactivity to sensory input. We can argue that such a peculiar pattern of psychopathological elements makes the identification of autistic features in this kind of patients more difficult. It is possible that the autistic traits have influenced the addictive pattern of our patient characterized by a rapid escalation of polysubstance use. Consistently, some authors showed maladaptive behaviors, including substance abuse and suicidal thoughts and behaviors, to be common in youths with ASD and associated with the presence of depression and PTSD, leading to suggest that individuals with ASD may represent a low-resilience group [11, 29]. Adults with ASD face substantial challenges accomplishing basic tasks associated with daily living, which are exacerbated by their broad and pervasive difficulties with social interactions. These challenges put people with ASD at increased risk for psychophysiological distress, which likely impacts social functioning for adults with ASD heavily, as suggested by growing literature on stress in children that indicates that those with ASD have differential responses to stress than healthy children [30]. In many such cases, there is a causal relationship between ASD and the comorbid psychiatric conditions in that the specific ASD symptoms result in chronic conflicts, misunderstandings, and failure in private and vocational relationships [31]. This may have accounted for the impact of the bullying the patient reported at the high school. A limitation in discussing this aspect is, however in this case, the lack of information on symptoms that may have occurred at that time. While detecting, in fact, the presence of symptoms of a developmental disorder such as ASD in adulthood, a priority for the clinician should be to report about patient's developmental history; however, it has been often reported how diagnostic procedure can be challenging due to a lack of accurate developmental information and a mixed clinical presentation when such patients visit psychiatric clinics for cooccurring psychiatric symptoms in adulthood. Further, although individuals with subthreshold ASD often report social adjustment, clinicians may overlook the social isolation while the underlying social awkwardness is often not addressed despite being potentially related to the bullying received. Difficulties in the relative clinical significance of these symptoms are thus still unclear and deserve further investigation particularly in adulthood. [32, 33] High-functioning ASD patients present inability to express their difficulties, due to their language restrictions and empathy deficits, and these can lead these people to behavioral deviances (often self- or hetero-destructive) that challenge their personal environment ending up in the pursuit of psychiatric help [34]. We believe that the patient had used substances to cope with everyday stressors [4, 11, 35]. The literature on the cooccurrence of SUD with ASD is scarce [15]. There are no methodologically sound studies, but clinical probing of families and professionals regarding their experiences with the cooccurrence of SUD and ASD showed substance-related problems to be common among both adolescents and adults with ASD [17]. About treatment choices, we have combined a standard treatment for BD, based on valproic acid and lithium carbonate [32, 36, 37], with an integrated treatment for alcohol and BDZ abuse, including disulfiram, nalmefene, and clonazepam; this latter was chosen as agonist substitution treatment for BDZ dependence, in virtue of his high potency and slow-onset, long lasting action [38–41]. We debate whether this patient belongs to a subgroup of well-adapted, double-diagnosis patients, where autism spectrum symptomatology, together with mood instability and reward sensitivity features, influences addictive manifestations. We propose this subgroup of patients would benefit from an integrated pharmacological treatment that considers all the psychopathological autistic dimensions implicated. The present case showed the dilemma of a subthreshold mood disorder in childhood that finally reached the threshold for a full-blown episode in adulthood (with polysubstance and behavioral addictions) versus a subsiding comorbidity between mood disorder with those related addictions and ASD. Thus, signs and symptoms of both a Bipolar Disorder and an ASD might run isolated or in clusters during the entire lifetime, often not reaching the threshold for a categorical diagnosis until adulthood. Assessment scales of both psychopathological domains may help the clinician to detect symptoms and signs formerly not considered. However, clinical judgment can be informed but not substituted using instruments that are based on the key features of a single disorder. The most crucial step is to promote the awareness of ASD signs and symptoms during the entire lifetime, with a better definition of clinical phenotypes using a dimensional approach. Adult psychiatry does not emphasize these issues, with a trend not to train psychiatrists to diagnose ASD. The clinical result is that the less severe, despite highly invalidating, ASD cases often remain underdiagnosed in adult psychiatric settings.

## Figures and Tables

**Table 1:** The patient's scores on the RAADS-R and AdAS Spectrum.

RAADS-R	Subdomains	

	*Language and communication*	9/24
	*Social relatedness*	54/126
	*Sensorimotor and stereotypies*	15/51
	*Circumscribed interests*	15/39

AdAS Spectrum	Subdomains	

	*Childhood and adolescence*	13/21
	*Verbal communication*	7/18
	*Nonverbal communication*	21/28
	*Empathy*	7/12
	*Inflexibility and adherence to routine*	21/43
	*Restricted interests and rumination*	16/21
	*Hyper/hyporeactivity to sensory input*	3/17
